# Correction: Evidence that Mediator is essential for Pol II transcription, but is not a required component of the preinitiation complex in vivo

**DOI:** 10.7554/eLife.32061

**Published:** 2017-09-18

**Authors:** Natalia Petrenko, Yi Jin, Koon Ho Wong, Kevin Struhl

Petrenko N, Jin Y, Wong KH, Struhl K. 2017. Evidence that Mediator is essential for Pol II transcription, but is not a required component of the preinitiation complex in vivo. *eLife* **6**:e28447. doi: 10.7554/eLife.28447.

Published 12, July 2017

In the published article, the table containing the list of strains and uploaded as Supplementary file 1 was an older version of the table and hence not completely correct. We have now replaced this with the correct table.

The article has been corrected accordingly.

